# Overexpression of *PpSnRK1α* in tomato enhanced salt tolerance by regulating ABA signaling pathway and reactive oxygen metabolism

**DOI:** 10.1186/s12870-020-02342-2

**Published:** 2020-03-26

**Authors:** Wen-Ru Wang, Jia-Hui Liang, Gui-Fang Wang, Mao-Xiang Sun, Fu-Tian Peng, Yuan-Song Xiao

**Affiliations:** College of Horticulture Science and Engineering; State Key Laboratory of Crop Biology, Shandong Agricultural University, Tai’an, 271000 Shandong China

**Keywords:** *PpSnRK1α*, ROS metabolism, ABA signaling, Salt tolerance

## Abstract

**Background:**

SNF-related Kinase 1 (SnRK1) is a key component of the cell signaling network. SnRK1 is known to respond to a wide variety of stresses, but its exact role in salt stress response and tolerance is still largely unknown.

**Results:**

In this study, we reported that overexpression of the gene encoding the α subunit of *Prunus persica SnRK1* (*PpSnRK1α*) in tomato could improve salt stress tolerance. The increase in salt stress tolerance in *PpSnRK1α*-overexpressing plants was found to correlate with increased *PpSnRK1α* expression level and SnRK1 kinase activity. And *PpSnRK1α* overexpression lines exhibited a lower level of leaf damage as well as increased proline content and reduced malondialdehyde (MDA) compared with wild-type (WT) lines under salt stress. Furthermore, *PpSnRK1α* enhanced reactive oxygen species (ROS) metabolism by increasing the expression level of antioxidase genes and antioxidant enzyme activities. We further sequenced the transcriptomes of the WT and three *PpSnRK1α* overexpression lines using RNA-seq and identified about 1000 *PpSnRK1α*-regulated genes, including many antioxidant enzymes, and these genes were clearly enriched in the MAPK signaling pathway (plant), plant-pathogen interactions and plant hormone signaling transduction and can respond to stimuli, metabolic processes, and biological regulation. Furthermore, we identified the transcriptional levels of several salt stress-responsive genes, *SlPP2C37*, *SlPYL4*, *SlPYL8*, *SlNAC022*, *SlNAC042,* and *SlSnRK2* family were altered significantly by *PpSnRK1α*, signifying that *SnRK1α* may be involved in the ABA signaling pathway to improve tomato salt tolerance. Overall, these findings provided new evidence for the underlying mechanism of *SnRK1α* conferment in plant salt tolerance phenotypes.

**Conclusions:**

Our findings demonstrated that plant salt stress resistance can be affected by the regulation of the *SnRK1α*. Further molecular and genetic approaches will accelerate our knowledge of *PpSnRK1α* functions, and inform the genetic improvement of salt tolerance in tomato through genetic engineering and other related strategies.

## Background

Currently, salt stress affects over 6% of the global area (more than 800 million hectares of land), and the infected area continues to increase [1]. In plants, high salt can affect the absorption of water and nutrients and reduce photosynthesis, thereby inhibiting plant growth and causing yield loss. And at the cellular level, high salt can disturb normal metabolism and cause “physiological drought”, ionic toxicity or causing complex secondary damages to proteins, nucleic acids, and other macromolecular substances [2].

Fundamentally, the salt tolerance of plants is conferred by tolerance genes. The isolation of novel tolerance genes and effective identification of salt-resistant germplasm are key strategies for improving salt tolerance in plants [3]. Recently, extensive genetic and molecular studies using loss-of-function mutants and overexpression lines have been carried out to investigate the molecular basis of salt tolerance in model plants and crop species. It has been reported that protein kinases of the mitogen activated protein kinase (MAPK), Calcineurin B-like-interacting protein kinase (CIPK) and SNF1-related protein kinases family(SnRKs) families and many transcription factors (TFs), such as NAC(NAM, ATAF1,2, and CUC2), AP2/ERF, MYB, WRKY, bZIP, were found to play important roles in environmental stress responses [4–12]. Therefore, screening for salt-tolerant genes via molecular studies is a necessity for protecting the plants against salt stress.

The structure and function of SnRK1 are similar to those of the yeast SNF1 and mammalian AMPK [13]. SnRK1 holoenzyme is a heterotrimer comprising a catalytic α subunit, a regulatory γ or βγ subunit, and β subunit as a scaffold connecting α and γ subunits [13]. Phosphorylation of the conserved threonine on the T-loop of the catalytic α subunit is required for SNF1/AMPK/SnRK1 to remain active [14–17].

SnRK1 is involved in sucrose and lipid metabolism in plants and can respond to stress signals under stress conditions, thereby regulating plant growth and development [18]. For example, increased SNF1 catalytic activity and phosphorylation of the conserved Thr-210 in the activation loop occur in yeast cells exposed to sodium or oxidative stress, indicating that similar to AMPK, SNF1 is activated in response to these stresses [19]. Moreover, it has been reported that SnRK1 activity is required for flooding resistance in Arabidopsis [20]. Likewise, SnRK1 is an essential kinase induced by autophagy under various stress conditions in Arabidopsis [21].

The sucrose nonfermenting-1-related protein kinases(SnRKs)family can respond to stress through regulating multiple signaling pathways via cascade amplification of the stress signal and initiate a stress response. The SnRKs family can be further subdivided into three subfamilies—SnRK1, SnRK2, and SnRK3—according to the differences in tertiary structures [22]. In Arabidopsis, SnRK2.2/SnRK2.4/SnRK2.6 are important regulators of ABA signaling in response to abiotic stress [23], and SnRK3 indirectly participates in ABA signaling by interacting with the regulatory factors in the ABA signaling pathway, thereby enhancing stress tolerance in plants. For example, ABI2 interacts strongly with SnRK3.11/SnRK3.13/SnRK3.15 to regulate the expression of salt stress-related genes, rendering the plants more sensitive to the stress signal and enhancing salt tolerance [24, 25]. In Arabidopsis, SnRK1 is negatively regulated by the ABA-insensitive 1 (ABI1) and protein phosphatase 2C A group (PP2CA) [26]. Therefore, we speculate that SnRK1 is involved in abiotic stress response via ABA signaling or other pathways.

Peach (*Prunus persica*) is a unique plant species, probably originated from China. It contains abundant valuable genetic resources that can be used to study and improve stress tolerance in plants [27]. In this study, the full-length *Prunus persica SnRK1α* (*PpSnRK1α*) sequence was obtained from *Prunus persica* (Linn.) Batsch. The wild-type (WT) and three transgenic tomato seedlings overexpressing *PpSnRK1α* (OE-1, OE-4, and OE-7) in WT were used. In order to study the possible regulatory mechanism of SnRK1 under salt stress, we compared the phenotype of the WT and transgenic tomato plants under salt stress and analyzed the physiological indexes, including the metabolic capacity of ROS, and ability to resist osmotic stress. Moreover, we sequenced the transcriptomes of the WT and three *PpSnRK1α* overexpression lines using RNA-seq and identified approximately 1000 differentially expressed genes (DEGs). We further examined the expression levels of salt stress-related genes that significantly correlated with SnRK1 and validated the interactions between SnRK1 and known ABA receptors, which provide evidence for a role of SnRK1 in salt stress response, signifying that SnRK1α may be involved in the ABA signaling pathway or reactive oxygen (ROS) metabolism to improve tomato salt tolerance. Overall, our results demonstrate that the overexpression of *PpSnRK1α* is beneficial for enhancing salt tolerance in plants.

## Results

### *PpSnRK1α* overexpression lines have higher SnRK1 activity and increased salt tolerance

**Fig. 1:**
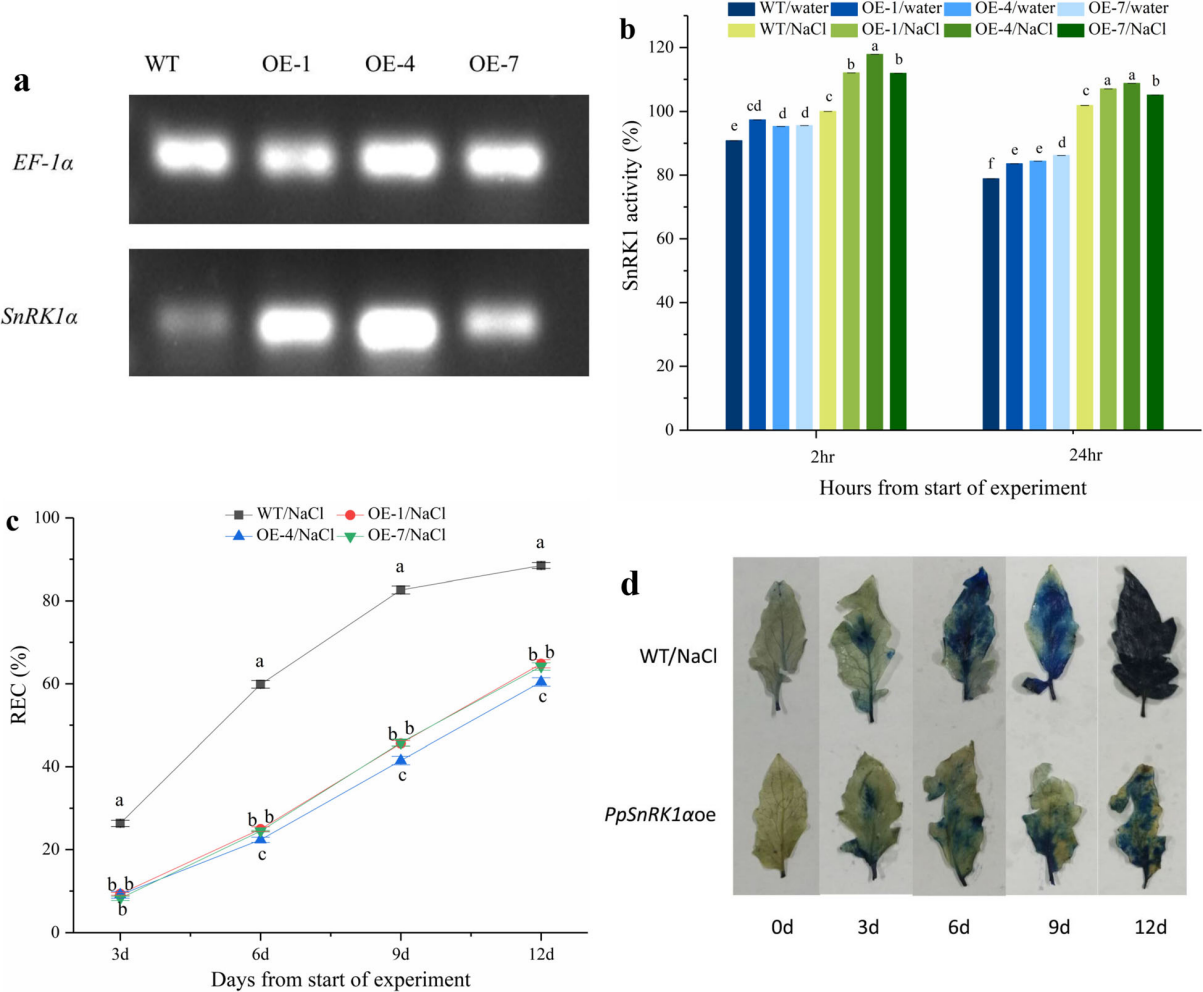
*PpSnRK1α* overexpression lines had higher SnRK1 activity and increased salt tolerance. **a** Relative expression level of *SnRK1* (PCR was performed using homologous and specific fragments of *PpSnRK1* (*Prunus persica*) and *SlSnRK1* (*Solanum lycopersicum*) between WT plants and *PpSnRK1α* overexpressing plants). This picture was cropped for simplicity and intuitiveness, the original pictures were in Additional file 1**b** Determination of SnRK1 activity **c** REC of the WT and *PpSnRK1α* overexpression lines **d** Evans blue stain. Note: in b and c, the data are represented as means ± standard error (S.E.) of three biological replicates

In order to further identify the difference between WT and *PpSnRK1α-*overexpressing tomatoes lines (*PpSnRK1α*oe), firstly we measured the expression levels of *PpSnRK1α* in overexpression lines (OE-1, OE-4, and OE-7) and WT. The results are shown in Fig. 1a. *PpSnRK1α*oe showed significant higher *SnRK1α* expression levels than WT, and *SnRK1α* expression levels were relatively higher in OE-1 and OE-4 than that of OE-7 (Fig. 1a, Additional file 1). Secondly, the SnRK1 activity of three *PpSnRK1α*oe significantly increased following two hours of the salt treatment and was 11.9–17.8% higher than that of the WT (Fig. 1b). However, SnRK1 activity declined after 24 h of high-salinity treatment both WT and *PpSnRK1α*oe. Thirdly, the relative electronic conductance of OE-1, OE-4, OE-7, and WT were 64.84, 60.43, 64.17, and 88.49%, respectively, after 12 days of salt treatment (Fig. 1c). In general, *PpSnRK1α* overexpression lines exhibited a lower level of cell damage than the WT. These observations were further validated by the Evans blue staining experiment (Fig. 1d). Thus, overexpression of *PpSnRK1α* increased the salt tolerance of plants, and the level of improvement was proportional to the level of *PpSnRK1α* gene expression and SnRK1 activity. Together, these results evidenced the role of *PpSnRK1α* overexpression in enhancing salt tolerance in tomato.

### *PpSnRK1α* regulates membrane lipid peroxidation and proline accumulation in response to salt stress

**Fig. 2:**
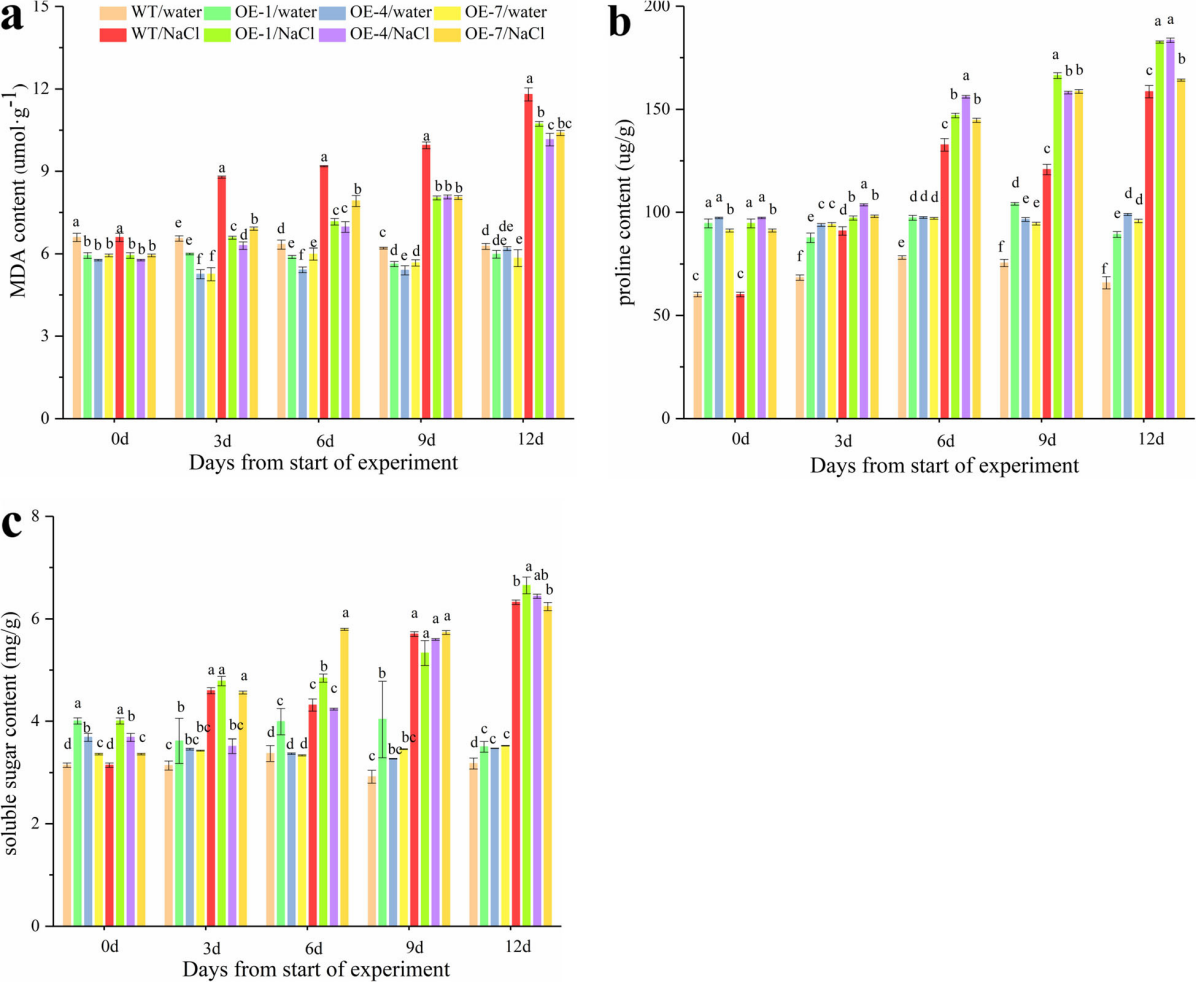
MDA, soluble sugar and proline contents in the WT and *PpSnRK1α* overexpression lines with and without the salt treatment. **a** MDA content **b** proline content **c** soluble sugar content. Three biological replicates were analyzed for each sample, and the data are represented as means ± S.E. of three technical repeats

To further determine if overexpression of *PpSnRK1α* attenuate the degree of peroxidation of the cell membrane and accumulate proline [28] to resist salt stress, we monitored changes in the MDA content and proline content in both WT and *PpSnRK1α*oe before and after the salt treatment. No significant difference in MDA content was observed between the *PpSnRK1α*oe and WT plants under normal condition, Whereas, the MDA content in *PpSnRK1α*oe was obviously lower than that in the WT after 12 days of high salinity treatment (Fig. 2a). On the other hand, *PpSnRK1α*oe exhibited a more significant increase of proline content than the WT plants after 12 days of control treatment (Fig. 2b). Furthermore, proline contents accumulated to higher levels in *PpSnRK1α*oe than in WT under salt stress (Fig. 2b). However, the total soluble sugar content also was determined and found that there was a limited difference in soluble sugar levels between *PpSnRK1α*oe and WT (Fig. 2c). Overall, these results indicate that *PpSnRK1α* overexpression leads to a decrease in the degree of cell membrane peroxidation and the accumulation of proline in response to salt stress.

### *PpSnRK1α* overexpression lines exhibit reduced ROS content and higher antioxidant enzyme activities under salt stress

To further investigate the mechanism of how *PpSnRK1α* overexpression lines enhances salt tolerance in tomato, the correlation between overexpression of *PpSnRK1α* and reactive oxygen metabolism in plants and the differences in antioxidant enzyme activities as well as ROS content between the WT and transgenic plants were studied. After 12 days of the salt treatment, the overexpression of *PpSnRK1α* resulted in a significant decrease (17–26%) in the O^2−^ content, compared with the level in WT plants (Fig. 2a). Consistent with these observations, NBT staining revealed that the transgenic leaves were less damaged than those of the WT under salt stress (Fig. 2b).

Under the normal growth condition, Superoxide dismutase (SOD), Catalase (CAT), Peroxisome (POD) activities of the transgenic lines were higher than those of the WT (Fig. 2c, e, f). Furthermore, the increases of SOD and POD activities in *PpSnRK1α* overexpression lines were much more evident, 62.9–96.7% and 8.0–15.8% increases than WT after 12 days of high salinity treatment (Fig. 2e, f). What’s more, *PpSnRK1α*oe exhibited higher CAT activity than the WT plants under salt stress. This result was consistent with those of the DAB staining assay, which indicated that transgenic plants have lower hydrogen peroxide content and higher CAT activity than WT (Fig. 2c, d). These results indicated that *PpSnRK1α* may play a role in antioxidant systems to protect plants under salt stress.

### Overexpression of *PpSnRK1α* alter the expression level of SnRK2 family genes (*SnRK2.1-SnRK2.7*) and antioxidase genes (*SOD*, *POD*, and *CAT1)* in tomato

The expression levels of *SOD*, *POD*, and *CAT1* by Quantitative Real-Time PCR (qRT-PCR) were detected both in *PpSnRK1α*oe and WT to further understand whether SnRK1 alters the expression of superoxide gene at the genetic level. According to Fig. 4, the transcript levels of genes encoding antioxidant enzymes (SOD, POD, and CAT) were the highest (6.839, 4.705 and 4.421 times than WT, respectively) among others in *PpSnRK1α* overexpression plants under high salinity condition (Fig. 4). Therefore, it can be seen that the overexpression of *PpSnRK1α* increased the expression level of the antioxidant enzyme genes at the genetic or transcriptional level.

There are seven *Solanum lycopersicum SnRK2* genes (*SlSnRK2s*) that are homologous to the Arabidopsis *SnRK2.2*/*SnRK2.3*/*SnRK2.6* genes in tomato [29]. In *PpSnRK1α* overexpression plants, the expression level of *SnRK2.1* is not significantly changed, and the expression of *SlSnRK2.2*, *SlSnRK2.3* and *SlSnRK2.5* were inhibited regardless of whether the salt stress was present or not (Fig. 4). By contrast, the expression level of *SlSnRK2.4* was increased in *PpSnRK1α*oe under both conditions (Fig. 4). However, the expression level of *SlSnRK2.7* was inhibited under normal conditions but increased under salt stress (Fig. 4)*.* Therefore, the overexpression of *PpSnRK1α* complexly affects the expression level of *SlSnRK2*.

### Transcriptomic analysis of WT and *PpSnRK1α* overexpression plants

To further evaluate the molecular mechanism of how *PpSnRK1α* overexpression confers salt tolerance on a broader scale, the differences in gene expression profile between three *PpSnRK1α*oe (OE-1, OE-4, and OE-7) and WT plants under normal condition were analyzed. Three biological replicates of the WT and three transgenic lines were sequenced using the Illumina platform, and approximately 40 to 51 million high-quality reads (with Fast QC quality score > 36) were obtained for each biological replicate (Additional file 2). Of all reads obtained, 92 to 94% could be mapped to a unique chromosomal position. The expression level of each transcript in the sample was represented by Fragments Per Kilobase of transcript per Million fragments mapped (FPKM). A high linear correlation was observed among the three replicates of the same sample, suggesting small differences among replicates (Additional file 3). Differentially expressed genes (DEGs) were identified by comparing the transcriptomes of the WT and transgenic plants [30, 31]. We identified a total of 1009 DEGs, among which 668 were upregulated and 341 were downregulated, in *PpSnRK1α* overexpression plants compared with the WT (Fig. 5a). These results revealed that the expression of a large number of genes was altered upon *PpSnRK1α* overexpression, and these genes are either directly or indirectly regulated by *SnRK1*.

**Fig. 3:**
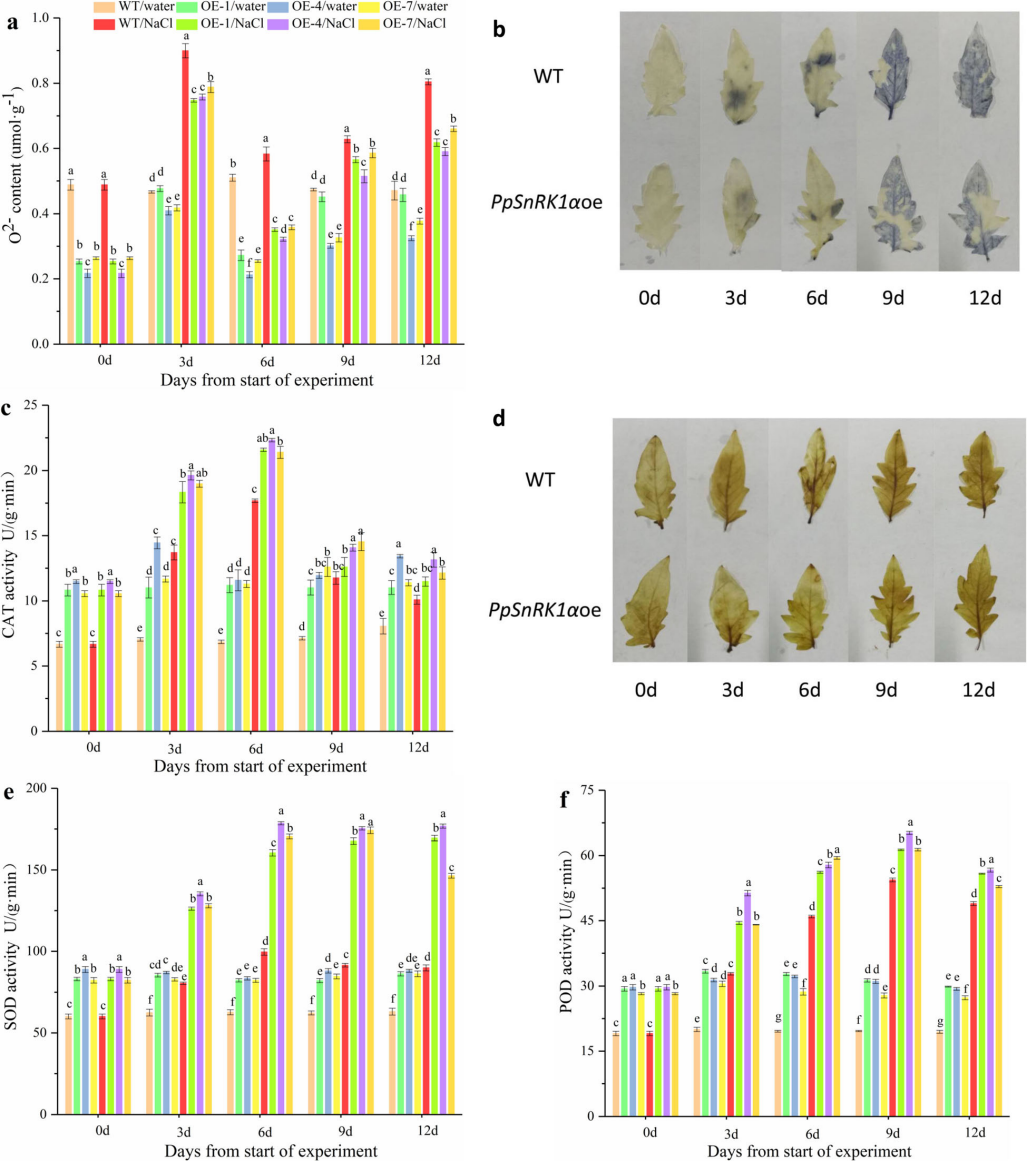
Antioxidant enzyme activities and the contents of O^2−^ in WT and *PpSnRK1α* overexpression lines. **a** Comparison of the O^2−^ content between the WT and *PpSnRK1α* overexpression lines, **b** NBT staining for superoxide in the leaves **c** Comparison of the CAT activity between the WT and *PpSnRK1α* overexpression lines **d** DAB staining for H_2_O_2_ in the leaves **e**, **f** Comparison of the SOD (e) and POD (f) activity between the WT and *PpSnRK1α* overexpression lines. Note: in a, c, e and f, each sample has three biological replicates. The data are shown as means ± S.E. of three technical repeats

To test whether *SnRK1α* overexpression relieves severe effects of salt by modulating the expression of salt-responsive genes, functional annotations of both up-regulated and down-regulated genes and KEGG pathway enrichment analysis in *PpSnRK1α*oe were performed (Additional file 4; Fig. 5b; Additional file 5). MAPK (mitogen activated protein kinase) signaling pathway–plant, plant-pathogen interaction, and plant hormone signal transduction were main KEGG pathways that were regulated in *PpSnRK1α*oe. Then GO terms were assigned to the DEGs. A number of genes were involved in various cellular component, molecular function, and biological processes (Fig. 5c), of which 1714 GO terms were enriched among the upregulated DEGs and 1334 GO terms among the downregulated DEGs (Additional file 6). Among the DEGs, response to stimuli, metabolic processes and biological regulation in biological process and antioxidant activity in molecular function were the obvious enriched GO terms (Fig. 5c). It is noteworthy that expression levels of 17 peroxidase genes were significantly regulated in *PpSnRK1α* overexpression plants, especially peroxidase 21 was up-regulated 6 times and may improve the ability of transgenic plants to remove ROS under salt conditions (Additional file 4). This hypothesis was supported by the patterns of ROS accumulation in the various lines (Fig. 3).

### *PpSnRK1α* overexpression alters the expression of several transcription factors and downstream genes, especially the transcript levels of stress-related genes

Of all the DEGs, we identified 101 transcription factors (TFs) that belong to 37 families (Additional files 7 and 8). Interestingly, the expression levels of TFs from the B3, GARP-G2-like, LOB, and WRKY families were upregulated, whereas those of TFs from other families, such as GRF (growth-regulating factors) and HSF (heat shock transcription factors), were suppressed. Given that over 100 TFs had altered expression levels upon *PpSnRK1α* overexpression, we concluded that the TFs were directly or indirectly affected by *PpSnRK1α*, and *PpSnRK1α* may regulate the expression of some genes or TFs by directly binding to their promoters.

To further investigate the role of SnRK1 in stress response, the expression levels of six stress-responsive genes between the WT and *PpSnRK1α*oe both two conditions were compared. Of the DEGs, there were genes encoding the stress-responsive protein, including phosphatase 2C (solyc03g096670.3), PYL (pyrabactin resistance(PYR)like) /PYR (pyrabactin resistance)(solyc06g050500.2 and solyc01g095700.3) involved in ABA signaling pathway [32].Surprisingly, the regulated TFs also included salt-related genes, such as *NAC* (No apical meristem domain-containing protein) family, some members of which have been shown to enhance the tolerance of rice and Arabidopsis to various abiotic stresses. As shown in Fig. 6a, RNA-seq showed that *SlPP2C37*, a member of the abiotic stress tolerance-related PP2C family, was significantly down-regulated under normal condition, and the expression levels of *SlPP2C37* was upregulated in *PpSnRK1α*oe and WT under salt stress, whereas the expression level of *SlPP2C37* was 1.3-fold lower than that in the WT plants (Fig. 6a, b). Furthermore, the expression levels of ABA-receptors *SlPYL4* (solyc06g050500.2) and *SlPYL8* (solyc01g095700.3) in *PpSnRK1α* overexpression plants were also altered (Fig. 6c, d, e, f). For example, the *SlPYL4* transcript level increased by 11 to 13-fold in *PpSnRK1α*oe under both normal condition and salt stress, which was consistent with the result of RNA-seq (Fig. 6c, d); whereas *PYL8* expression declined in the transgenic lines under normal condition consistent with the result of RNA-seq and slightly increased under salt stress (Fig. 6e, f). Interestingly, *SlNAC022* and *SlNAC042* were significantly upregulated higher in *PpSnRK1α*oe than that in WT under both normal condition and salt stress, and the up-regulated expression levels of *SlNAC022* and *SlNAC042* in the *PpSnRK1α*oe were 359.1-fold and 52.0-fold, respectively, higher than those in the WT under salt stress (Fig. 6g, h, i, j). These data indicated that the overexpression of *SnRK1α* could alter the expression of stress-related genes, and had the potential for improving the salt tolerance of tomato plants.

## Discussion

When plant responses to salt stress, some protein kinases are activated, which then regulates a large number of defense-related genes, and these changes are conducive to the establishment of defense mechanisms in plants. The response of plants to salt stress is closely related to the SnRKs family, which can be through the SOS (Salt Overly Sensitive) signaling pathway and ABA signal transduction [2]. Although it is unclear how plants respond specifically to stress, there is ample evidence that SnRKs can participate in resistance to salt stress in many species [18, 33, 34].

As a key component of the cell signaling network, *SnRK1* can interact with sugar molecules of different forms and energy states, regulate the activities of other kinases, and interact with TFs to maintain energy balance within the cell under stress conditions [18]. To our knowledge, *AKIN10*(homologous with *PpSnRK1α*) can negatively regulate the accumulation of AtMYC2 protein via AKIN10 kinase activity-dependent protein modification, thereby responding to abiotic stress [35]. *SnRK1* also controls Na^+^ flux and maintains Na/K homeostasis under high salinity stress [36]. Moreover, *SnRK1* can respond to high or low glucose levels in the dark as well as under hypoxia and saline conditions, and regulate energy metabolism in plants under stress [37]. In this study, we identified *PpSnRK1α* and showed that ectopically expressing it in tomato enhanced salt tolerance phenotypes (Fig. 1). Consistent with previous studies, overexpressing *PpSnRK1α* lines had higher cell viability and grew better under salt stress, which was accompanied by higher *PpSnRK1α* expression level and SnRK1 activity (Fig. 1), indicating that *SnRK1* is a positive regulator of salt tolerance in tomato. Therefore, *PpSnRK1α* may have potential additional mechanisms in addition to its role in salt tolerance.

**Fig. 4:**
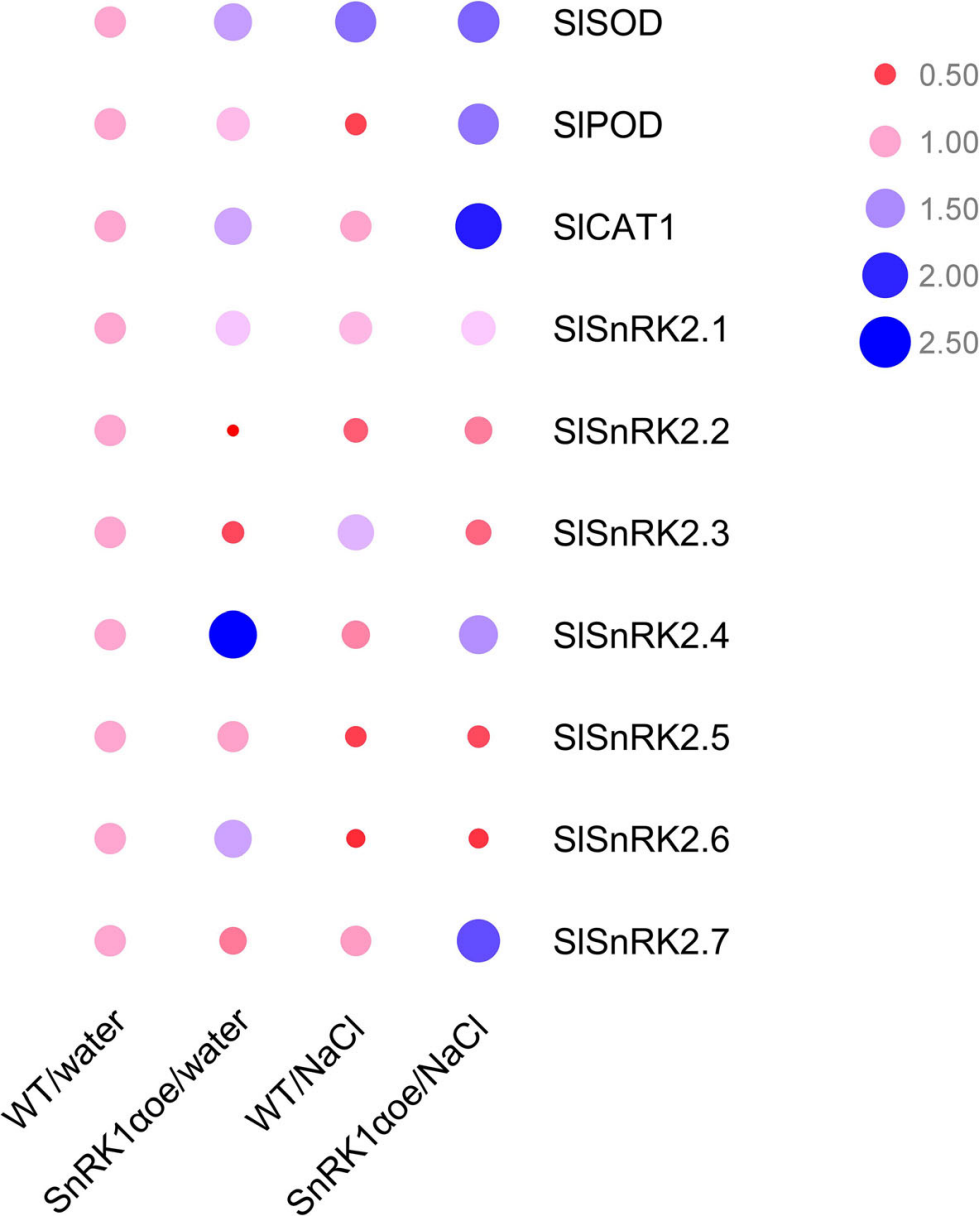
qRT-PCR analysis of the relative expression levels of *SlSOD*, *SlPOD*, *SlCAT1* and *SlSnRK2* genes (*SlSnRK2.1*- *SlSnRK2.7*) in the WT and *PpSnRK1α* overexpression lines

Accumulation of reactive oxygen species is one of the main responses of plants to salt stress. Excessive ROS will destroy biological macromolecules and produce toxic effects on cells. Several antioxidant enzymes in plants, such as antioxidant dismutase, peroxidase, and catalase, play critical roles in the process of scavenging ROS [38]. It was reported that ROS could also be used as a signaling molecule to mediate plant responses to different stresses [39, 40]. Some protein kinases in the cell can sense the stimulation of exogenous ROS or the production of ROS, and respond to stress through a series of phosphorylation and dephosphorylation signals. For example, NADPH oxidase can rapidly increase the intracellular ROS level, and the stimulus signal quickly enters the nucleus from the plasma membrane of the cell [41]. Szymańska, P. Katarzyna et al. found that two SNF1-releted protein kinases 2 (SnRK2), SnRK2.4 and SnRK2.10, could regulate ROS homeostasis and respond to salinity in Arabidopsis [42]. In our investigation, overexpressing *PpSnRK1α* lines had increased ROS scavenging capacity, low degree of cell membrane damage, and remarkably higher expression of antioxidant enzyme genes under salt stress, which could partially explain the mechanism responsible for the improved salt tolerance of the transgenic plants and suggest a role of *PpSnRK1α* in regulating the expression of antioxidase genes (Figs. 2, 3, 4, and 7). In general, proline is considered to be one of the osmotic regulators in wheat to resist salt stress, and the accumulation of proline help maintain the integrity and function of the cellular vacuolar membrane, thereby enhancing its ability to resist salt damage [43, 44]. It is apparent that *PpSnRK1α* helps to maintain the integrity and function of cell membranes, thus enhancing the plant’s ability to resist salt damage (Fig. 2). Therefore, we think that these may have contributed, at least partially, to the observed salt tolerance.

The *SnRKs* represent a large family that can be further divided into the *SnRK1*, *SnRK2,* and *SnRK3* subfamilies. Many members of the *SnRKs* family have been shown to play key roles in abiotic stress responses. AREB1/ABF2, AREB2/ABF4, and ABF3 are activated by SnRK2.2, SnRK2.6 andSnRK2.3 in ABA signaling in response to osmotic stress during vegetative growth [45]. SnRK2.2 and the closely related SnRK2.3 are known to play roles in ABA inhibition of seed germination in Arabidopsis [46]. Additionally, SnRK2.6 can interact with ABAR/CHLH and response to abscisic acid in guard cell signaling [23], The results showed that when plants were under salt stress, the overexpression of *PpSnRK1α* significantly affected the expression levels of seven *SnRK2* family genes in tomato (Fig. 4). Moreover, the research about the SnRKs family revealed that there are also interactions between different genes in the family. For example, in Arabidopsis, SnRK3.15 can interact with SnRK1.1 and SnRK1.2, regulate the activity of them and participate in sugar metabolism [47]. Therefore, the direct or indirect interaction between members of different subfamilies of the plant SnRKs strengthens the plant’s ability to respond to stress.

**Fig. 5:**
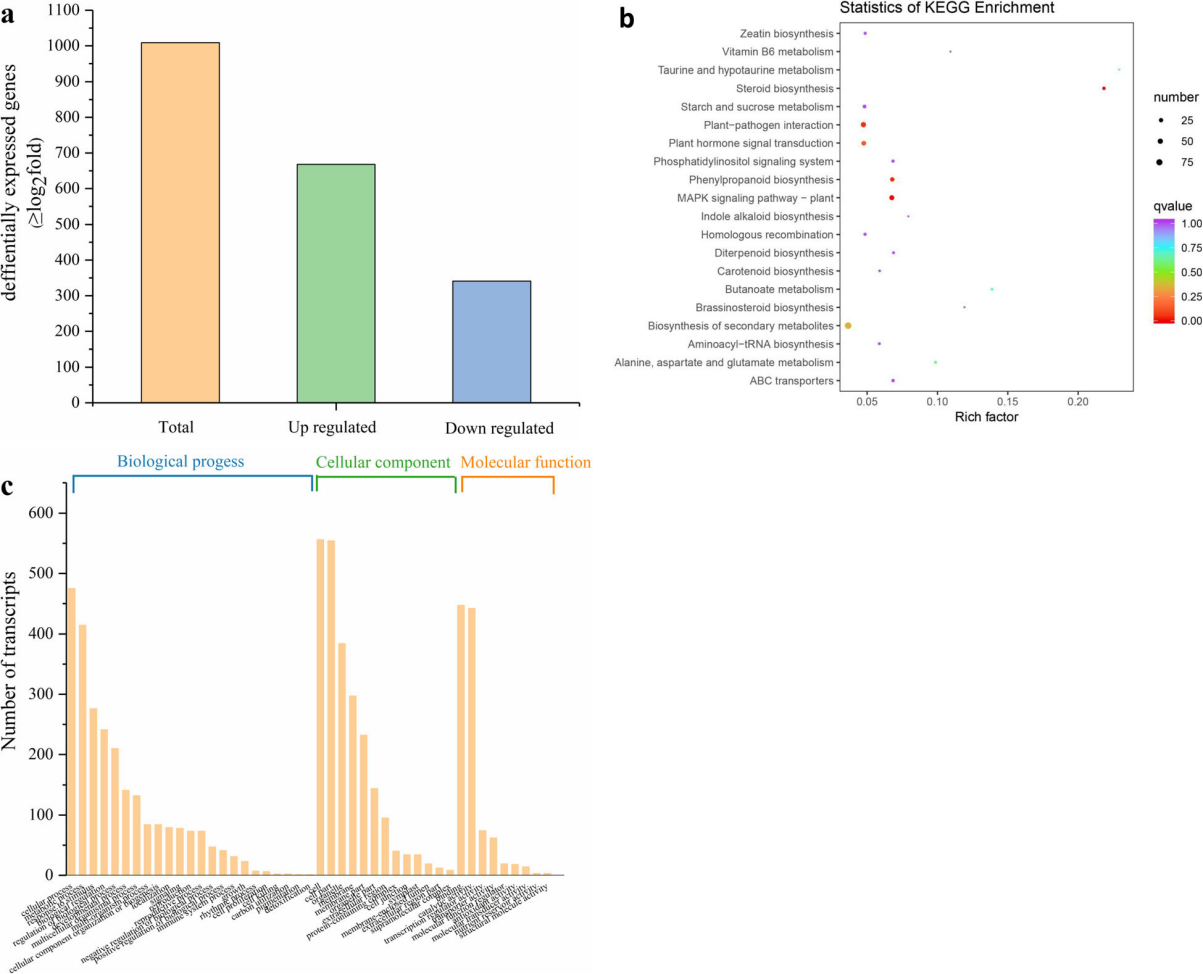
Identification and functional annotation of the DEGs. **a** DEG identification. **b** KEGG classification analysis of the DEGs **c** GO classification analysis of the DEGs

**Fig. 6:**
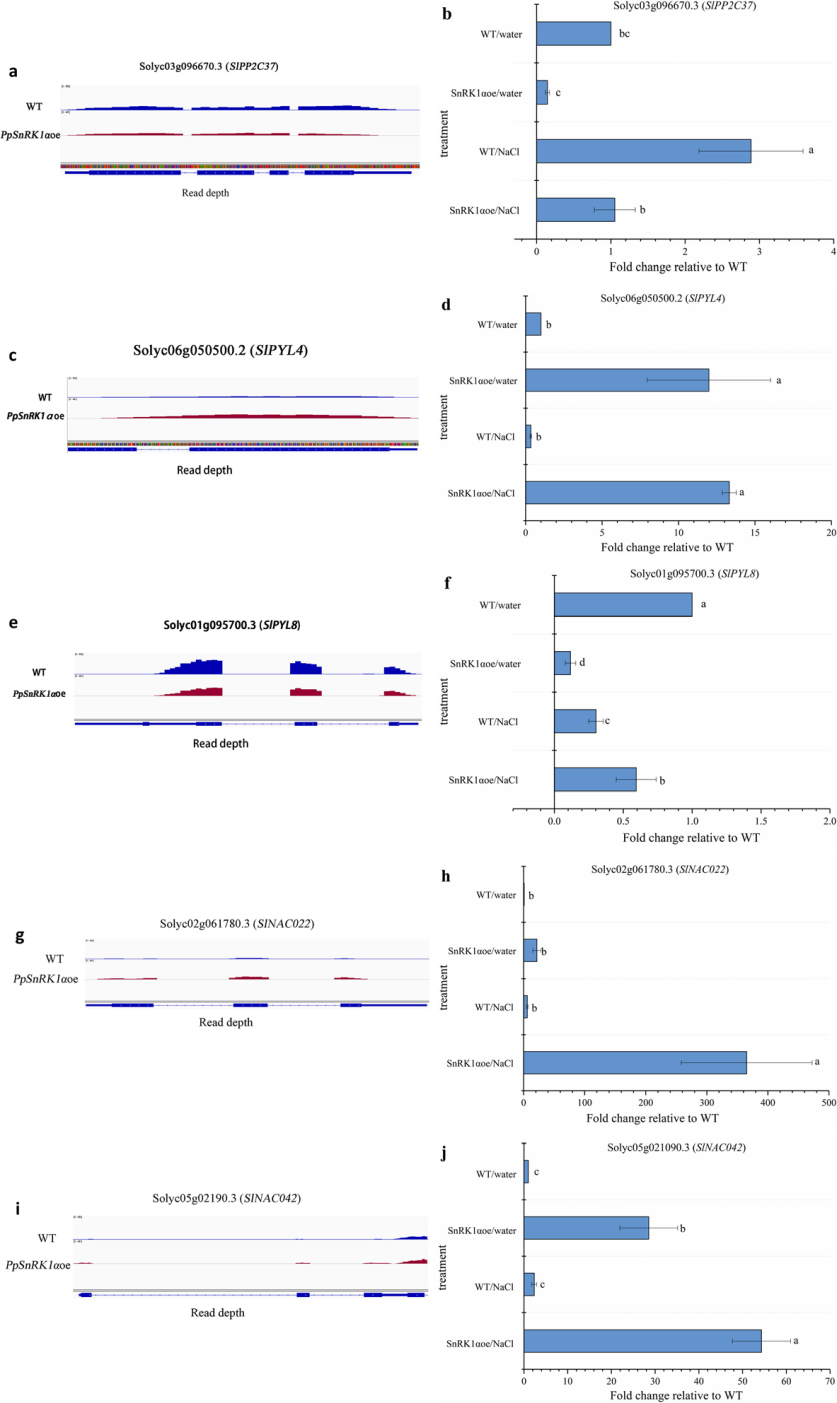
IGV (Integrative Genomics Viewer) visualization of genes by RNA-seq and results of RT-qPCR analysis of related genes **a**, **c**, **e**, **g** and **i** IGV visualization of *SlPP2C37* (a), *SlPYL4* (c), *SlPYL8*(e), *SlNAC022* (g)and *SlNAC042*(i) **b**, **d**, **f**, **h** and **j** Results of RT-qPCR analysis of *SlPP2C37* (b), *SlPYL4* (d), *SlPYL8*(f), *SlNAC022* (h)and *SlNAC042*(j). Note: in b, d, f, h and j, error bars indicate SEs (*n* = 3, 3 biological replicates). All values for WT and *PpSnRK1α* overexpression plants are statistically significantly different from each other (Student’s t-test; *p*-value < 0.05)

**Fig. 7:**
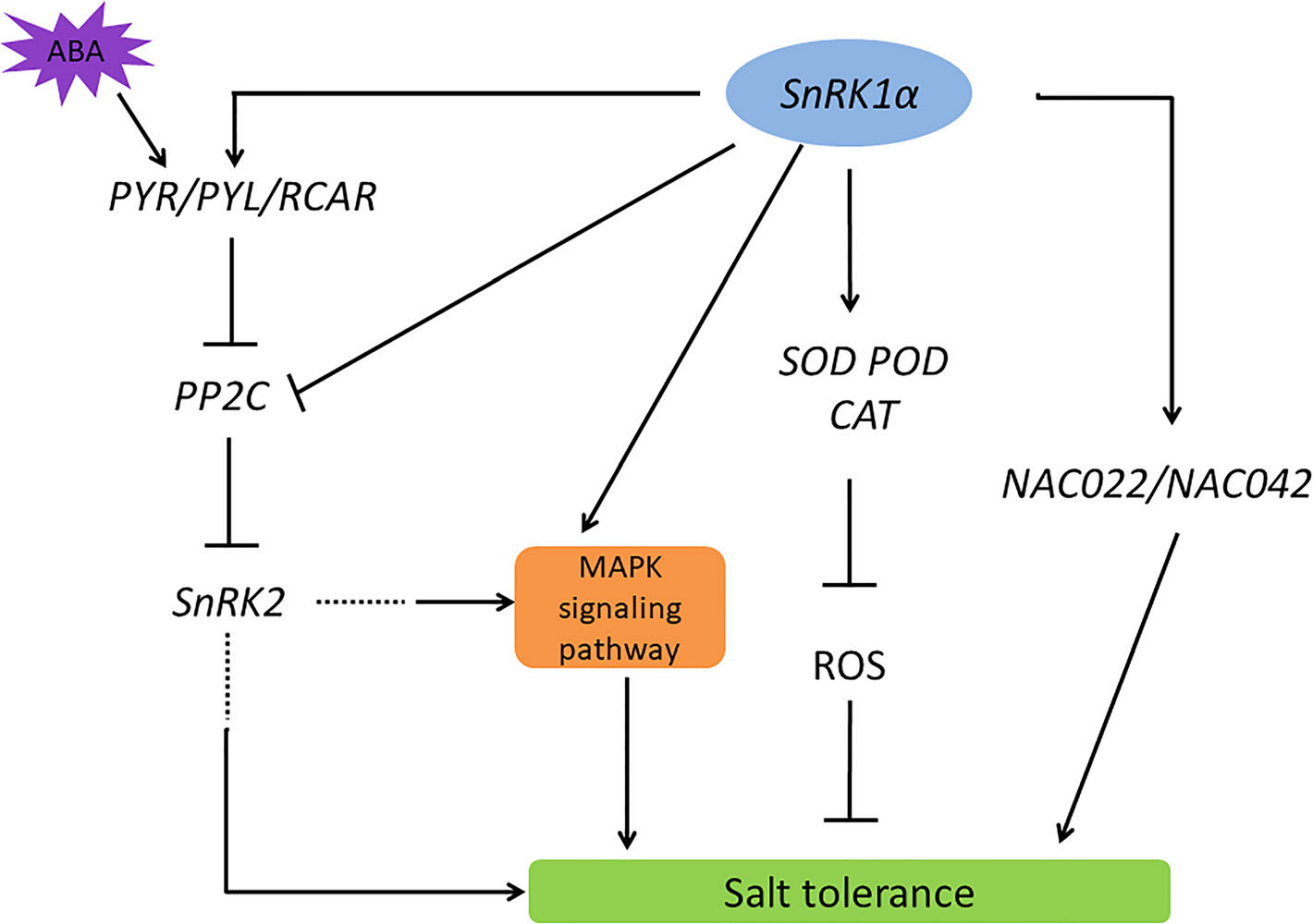
The working model of *PpSnRK1α* regulating salt tolerance through the ABA signal transduction pathway and ROS mechanism. In the current working model, *PpSnRK1α*-overexpression improves the transcription levels of *SOD*, *POD,* and *CAT1* as well as SOD, POD and CAT activity, reduces ROS production in plant cells. *PpSnRK1α* is involved in the ABA signaling pathway by altering the transcription levels of ABA receptors (*SlPYL4*, *SlPYL8*), PP2C (*SlPP2C37*) and *SlSnRK2s*, and thereby leads to an increase in tolerance to salt stress in tomato. Additionally, *PpSnRK1α*-overexpression increases the expression of *SlNAC022*, which has been proved to participate in ABA signaling transcription

Abscisic acid is a key player in regulating abiotic stress response in plants. A previous study has evidenced the interaction between *SnRK1.1* and ABA signaling—the crossing of an *SnRK1.1* overexpression Arabidopsis line with the *aba2* mutant, which was impaired in both ABA signaling transduction and sugar signaling pathway, led to glucose hyposensitivity [48]. In addition, defective kernel 33 (DEK33) is involved in the ABA synthesis process and can interact with SnRK1, suggesting that their interaction may regulate the stability of DEK33 and thus the ABA signal [49]. It was reported that inhibition of *SnRK1* expression in peas resulted in pleiotropic maturation defects similar to those of the ABA insensitive mutants and SnRK1 can respond to ABA treatment in wheat roots, the level of which drops sharply, but the amount of phosphorylated (active) SnRK1 remains constant [50, 51]. In our study, we found that the expression of *SlPP2C37* (a member of PP2C family) [26], *SlPYL4*, *SlPYL8* (an ABA receptor in ABA signaling) and *SnRK2* family genes were significantly regulated upon *PpSnRK1α* overexpression under the normal growth condition as revealed by the RNA-seq data (Figs. 5, 6 and 7). MAPK (mitogen activated protein kinase) cascades have been shown to be implicated in ABA signaling and abiotic stress as well [32]. The RNA-seq results showed that the MAPK pathway was significantly regulated in the *PpSnRK1α* overexpression lines (Fig. 5, Additional file 4). These genes have been shown to be involved in the ABA signaling pathway [5]. According to these results, we presumed that *SnRK1α* may be involved in the ABA signal transduction pathway. Further verification is needed to illustrate the regulatory mechanism of *SnRK1α* under salt stress.

NAC proteins, as one of the largest family of transcription factors unique to plants, are known to possess diverse roles in plant response to environmental stress [52]. It was reported that *ANAC036* (NAC transcription factor family member in Arabidopsis) involved in inflorescence and leaf morphogenesis in Arabidopsis [53]. And Hong Y et al. found that *ONAC22* (NAC transcription factor family member in rice) played a positive role in drought and salt stress tolerance through modulating an ABA-mediated pathway [54]. Our investigation showed that *SlNAC022*, which has high homology with *ANAC036* and *ONAC22*, was significantly regulated in *PpSnRK1α* overexpressing plants (Fig. 6). Overexpression of JUNGBRUNNEN1 (*JUB1*) can strongly delay senescence and enhanced tolerance to various abiotic stresses in Arabidopsis [55, 56]. Interestingly, the expression level of *SlNAC42*, which has high homology with *JUB1*, is also significantly higher than that in WT plants (Fig. 6). Here we hypothesize that the up-regulation of *SlNAC022* and *SlNAC42* in *PpSnRK1α* overexpressing leaves enhances plant salt tolerance in response to ABA signals. More work is required to further determine if SnRK1 and NAC022/ NAC42 interact directly to improve salt tolerance.

Here we showed that over-expression of *PpSnRK1α* improved the salt tolerance of plants. These observations indicated that stress resistance could be affected by the regulation of the *PpSnRK1α* gene. Further molecular and genetic analysis will increase our understanding of the function of *PpSnRK1α* in plant stress resistance and provide strategies for breeding transgenic crop varieties with high-resistance.

## Conclusions

In summary, the results of this study indicate that SnRK1 functions as a key kinase for stress response and *PpSnRK1α* overexpression can significantly improve salt tolerance via regulating ROS metabolism or possibly ABA-mediated pathways. What’s more, SnRK1 may direct or indirect interacts with the SnRK2 family. The mechanism by which SnRK1 responds to salt stress is complex, and whether SnRK1 directly regulates *NAC022* and *NAC042* expression or indirectly controls their transcription by bind to intermediate receptors warrants further investigations. Generating *snrk1* loss-of-function mutants might be necessary to further understand the molecular mechanisms underlying the function of *SnRK1* in salt stress response and tolerance.

## Methods

### Vector construction and tomato transformation

In this study, the WT tomato (*Solanum lycopersicum* Mill. cv. Zhongshu 6) were kindly provided by Qing-wei Meng [57]. Then we obtained over-expressing *PpSnRK1α* tomatoes by vector construction and tomato transformation, and selected three lines (OE-1, OE-4, and OE-7) for further research.

Based on the coding sequence of *PpSnRK1α* (https://www.ncbi.nlm.nih.gov/nuccore/XM_007215174.2), two primers were designed for the amplification (forward primer: 5′- GCTCTAGAATGGATGGATCGGTTG-3′; reverse primer: 5′- GCGTCGACTTAAAGGACCCG − 3′). Then, the *PpSnRK1α* coding sequence was inserted in the sense orientation into the binary vector pBI121 downstream of the 35S promoter of the cauliflower mosaic virus (35S: *PpSnRK1α*). The construct was introduced into the *Agrobacterium tumefaciens* strain LBA4404 for tomato (*Solanum lycopersicum* Mill. cv. Zhongshu 6) transformation using the leaf disc method [58].

T2 transgenic plants overexpressing *PpSnRK1α* were identified by the T5 direct PCR kit (TSINGKE, TSE011) using primers *PpSnRK1α*-DF (5′-GTGTGGAGTTGCGGAGTCAT-3′) and *PpSnRK1α*-DR (5′-ACGAGGAAGATGAGCCTGGA-3′). The PCR-positive tomato plants were transplanted into pots with soil and grown under natural conditions. Next, we selected three transgenic tomato lines (OE-1, OE-4, and OE-7) for further research.

### Plant materials and treatments

The WT and transgenic tomato seeds were cultured for 4 weeks in the greenhouse at 25 ± 3 °C under a 16 h light /8 h dark cycle. After 1 month, the WT and the three transgenic lines, OE1, OE4, and OE7, were treated by either water (the control treatment) or 100 mmol·L^− 1^ sodium chloride (NaCl). Each treatment contained three groups with ten seedlings per group. Salt stress was introduced by watering the plants with 1000 mL 100 mmol·L-1 NaCl solution until saturation every 3 days. The leaves samples were collected at days 0, 3, 6, 9 and 12 after the treatment. SnRK1 enzyme activity was measured two and 24 h following the NaCl treatment on day one.

### RT-PCR

Total RNA was extracted from tomato leaves using Ultrapure RNA Kit (CWBIO, China, CW0581M) and reverse transcribed into cDNA using PrimeScript™ II 1st Strand cDNA Synthesis Kit (Takara, 6210A). The volume of each cDNA pool was adjusted to give the same PCR signal strength for *EF1α* after 32 cycles. PpSnRK1 and SlSnRK1 homologous segments were selected to design specific primers (*PpSlSnRK1α*-F and *PpSlSnRK1α*-R) for PCR identification (Additional file 9). PCR was carried out using the Premix Taq (Takara, R004A).

### SnRK1 activity determination

One gram of each fresh plant sample harvested at two and 24 h after the salt treatment, was ground in 1 mL of cold extraction buffer containing 100 mM Tricine-NaOH (pH 8.0), 25 mM NaF, 5 mM dithiothreitol, 2 mM tetrasodium pyrophosphate, 0.5 mM ethylene diamine tetra acetic acid, 0.5 mM ethylene glycol tetra acetic acid, 1 mM benzamidine, 1 mM phenylmethylsulfonyl fluoride, 1 mM protease inhibitor cocktail (Sigma P9599), phosphatase inhibitors (PhosStop; Roche), and insoluble polyvinylpyrrolidone to the solution with a final concentration of 2% (w/v). The homogenate was transferred to two cold microfuge tubes and centrifuged at 12,000×*g* for 5 min at 4 °C. The supernatant (750 uL) was desalted on a 2.5 mL centrifuge column (Sephadex G-25 medium columns; GE Healthcare) that was pre-equilibrated. SnRKl activity was determined by the Universal Kinase Activity Kit (R&D Systems, Minneapolis, MN, United States, EA004) by using AMARA polypeptide as the substrate [59].

### Determination of antioxidant capacity and the content of proline

Proline content was evaluated by the acid ninhydrin method [60]. The O^2−^ content was quantified by the hydroxylamine oxidation method [61]. 3,3′-diaminobenzidine (DAB) staining and nitroblue tetrazolium (NBT) staining were performed to detect H_2_O_2_ and O^2−^ levels in tomato leaves. Specifically, tomato leaves were first immersed in a 0.5 mg/mL DAB staining solution (pH = 3.8) or 0.5 mg/ml NBT solution, and then placed in a dark, high-humidity plastic box for about 24 h. Next, the staining solutions were decanted and the leaf samples were incubated with an ethanol/lactic acid/glycerol solution (3:1:1, v:v:v) in a boiling water bath for 5 min [62]. The SOD activity was determined by the photochemical reduction method using nitroblue tetrazolium [63]. The CAT activity was determined by the ultraviolet absorption method and change in the optical density at 240 nm (OD240) of 0.1 within 1 min was defined as one unit of enzyme activity [64]. POD activity was measured by the guaiacol method at OD470, and one unit enzyme activity was defined as 0.1 decease in OD470 per minute [65]. SOD, POD, and CAT activities are shown as U·min^− 1^·g^− 1^ (FW).

The degree of damage to the cells was determined by measuring the relative conductivity of tomato leaves. Ten leaf disks (0.8 cm in diameter) from the WT or transgenic lines were submerged in 20 mL distilled water, vacuumed for 30 min, shaken for 3 h at room temperature, and measured for the initial electric conductivity (S1). Subsequently, these leaf samples were boiled for 30 min and cooled down to room temperature to measure the final electric conductivity (S2). The electric conductivity of pure distilled water was used as the blank (S0). The relative electronic conductance (REC) was calculated as $\mathrm{REC}=\frac{\mathrm{S}1-\mathrm{S}0}{\mathrm{S}2-\mathrm{S}0}\ast 100$. MDA content was determined by the thiobarbituric acid method [66]. Tomato leaves were stained with the Evans blue staining solution to assess cell viability [67].

### RNA-seq

Leaves of WT and three *PpSnRK1α* overexpressing lines of tomato grown under normal conditions for 2 months were collected. RNA isolation, quality control, library construction, and Illumina Hiseq sequencing were performed by the Wuhan Metware Biotechnology co., LTD.

### Bioinformatic analysis

#### Quality control

Raw reads were removed low-quality reads and reads containing adapters and poly-N from the raw data, then filtered and examined for the sequencing error rate and GC content. The resulting clean, high-quality reads were used for subsequent analyses.

#### Mapping and differentially expressed gene (DEG) identification

The sequences were mapped to the tomato genome using HISAT2. The FPKM and count value were calculated using feature Counts. DESeq2 was used to analyze the differences between different samples and treatment groups [30, 31]. Then Multiple hypothesis test correction was performed using the Benjamin-Hochberg method at a significance level of *P* < 0.05; |log 2 fold change| ≥ 1 was used to identify differentially expressed genes (DEGs) between the two libraries (*PpSnRK1α*oe and WT). The Gene Ontology (GO) and Kyoto encyclopedia of Genes and Genomes (KEGG) enrichment analyses of the DEGs were performed using R based on the hypergeometric distribution.

#### qRT-PCR

Quantitative Real-Time PCR (qRT-PCR) was carried out using the UltraSYBR mixture (CWBIO, China, CW2601M). The 25 μL reaction mixture contained 0.5 μL of forward primer (10 μM), 0.5 μL reverse primer (10 μM), 5 ng cDNA template, 12.5 μL 2× UltraSYBR mixture, and 9.5 μL water. The conditions used for qRT-PCR were as follows: 95 °C for 10 min, followed by 40 cycles of 95 °C for 10 s, 60 °C for 30 s, and 72 °C for 32 s. The calculation method for qRT-PCR is 2^-ΔΔCt^ with the *EF-1α* gene as the endogenous control. Each sample had three independent biological replicates. Refer to Additional file 8 Table S6 for primers used for RT-PCR.

### Statistical analysis

Statistical analysis was performed using Microsoft Office Excel 2010 and images were processed with Microsoft PowerPoint 2010. Comparison of the mean values was performed by the Duncan multi-range test in SPSS 20.0. P < 0.05 denotes significant differences.

## Supplementary information

**Additional file 1 : Figure S1.** Relative expression level of *SnRK1α* (the original, uncropped gel).

**Additional file 2 : Table S1.** Mapping statistics of RNA-seq reads.

**Additional file 3 : Figure S2.** Scatter plot of FPKM values between replicates or genotypes.

**Additional file 4 : Table S2.** List of DEGs and their expression levels in WT and *PpSnRK1α* overexpression lines.

**Additional file 5 : Table S3.** KEGG enrichment analysis.

**Additional file 6 : Table S4.** GO enrichment analysis.

**Additional file 7 : Figure S3.** 101 TFs of 37 families regulated by *PpSnRK1α.*

**Additional file 8 : Table S5.** List of TFs and their expression levels in WT and *PpSnRK1α* overexpression lines.

**Additional file 9 : Table S6.** primers used in this study.

## Data Availability

The datasets supporting the conclusions of this article are included within the article and its additional files. The coding sequence of *PpSnRK1α* sequence is available at NCBI (https://www.ncbi.nlm.nih.gov/nuccore/XM_007215174.2).

## Abbreviations

WT: Wild-type
PpSnRK1α: SNF-related Kinase 1α subunit (*Prunus persica*)
*PpSnRK1α*oe: *PpSnRK1α*-overexpressing tomatoes lines
ROS: Reactive oxygen species
qRT-PCR: quantitative real-time PCR
ABA: Abscisic acid
REC: Relative electronic conductance
DEGs: Differentially expressed genes
FPKM: Fragments per kilobase of exon per million fragments mapped
GO: Gene ontology consortium
KEGG: Kyoto encyclopedia of genes and genomes
MAPK: Mitogen activated protein kinase
CIPK: Calcineurin B-like-interacting protein kinase
ABI1: ABA-insensitive 1
PP2CA: Protein phosphatase 2C A group
TFs: Transcription factors
GRF: Growth-regulating factors
HSF: Heat shock transcription factors
PYL/PYR: Pyrabactin resistance(PYR)like/ pyrabactin resistance
NAC: NAM, ATAF1,2, and CUC2
SOS: Salt overly sensitive
