# Supplementary material for: Overexpression of PpSnRK1α in tomato enhanced salt tolerance by regulating ABA signaling pathway and reactive oxygen metabolism
Source: BMC Plant Biol. 2020 Mar 26;20:128. doi: 10.1186/s12870-020-02342-2 (PMC7099830; doi:10.1186/s12870-020-02342-2)
Supplement: Supplementary file 1 — Additional file 1 : Figure S1. Relative expression level of SnRK1α (the original, uncropped gel). [file 12870_2020_2342_MOESM1_ESM.docx]

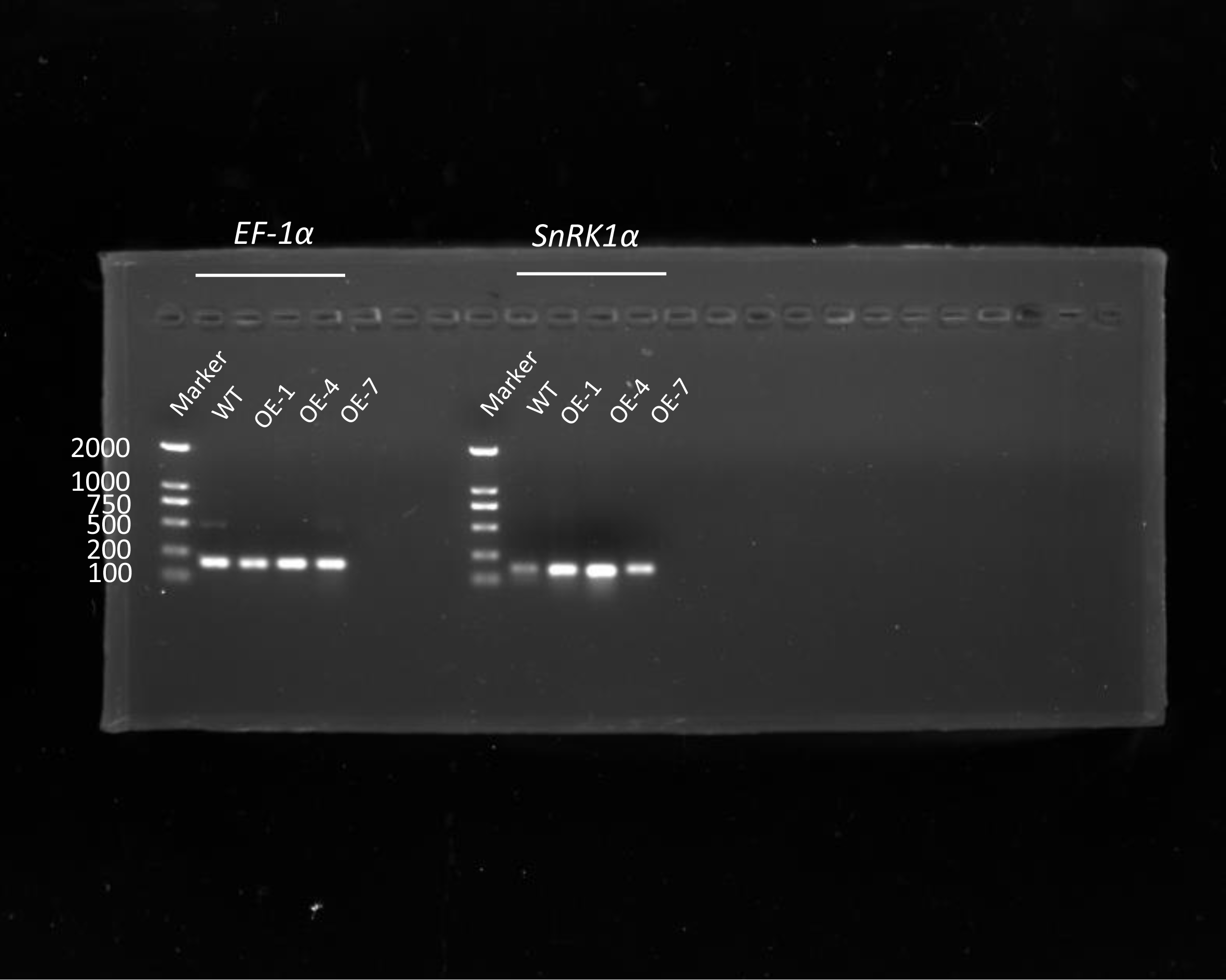


Figure S1 Relative expression level of *SnRK1α* (the original, uncropped gel of figure 1a). The expression levels of *SnRK1α* in WT, OE-1, OE-4, and OE-7 were detected by PCR, *EF-1α* was used as a control. 10 ul of each PCR product was taken for agarose gel electrophoresis.
