# Supplementary material for: Overexpression of PpSnRK1α in tomato enhanced salt tolerance by regulating ABA signaling pathway and reactive oxygen metabolism
Source: BMC Plant Biol. 2020 Mar 26;20:128. doi: 10.1186/s12870-020-02342-2 (PMC7099830; doi:10.1186/s12870-020-02342-2)
Supplement: Supplementary file 2 — Additional file 2 : Table S1. Mapping statistics of RNA-seq reads. [file 12870_2020_2342_MOESM2_ESM.docx]

Table S1:Mapping statistics of RNA-seq reads

| Sample | Total Reads | Reads mapped | Unique mapped | Multi mapped |
| --- | --- | --- | --- | --- |
| WTL13 | 46110852 | 44007303(95.44%) | 43314956(93.94%) | 842303(1.50%) |
| WTL14 | 41909954 | 40292590(96.14%) | 39670600(94.66%) | 746180(1.48%) |
| WTL15 | 54195414 | 52239207(96.39%) | 51388757(94.82%) | 1028803(1.57%) |
| OELS16 | 46952416 | 45023750(95.89%) | 44284883(94.32%) | 915616(1.57%) |
| OELS17 | 47676366 | 45710600(95.88%) | 44963493(94.31%) | 925590(1.57%) |
| OELS18 | 47105434 | 44333866(94.12%) | 43636114(92.63%) | 865574(1.48%) |
