# Supplementary material for: Overexpression of PpSnRK1α in tomato enhanced salt tolerance by regulating ABA signaling pathway and reactive oxygen metabolism
Source: BMC Plant Biol. 2020 Mar 26;20:128. doi: 10.1186/s12870-020-02342-2 (PMC7099830; doi:10.1186/s12870-020-02342-2)
Supplement: Supplementary file 3 — Additional file 3 : Figure S2. Scatter plot of FPKM values between replicates or genotypes. [file 12870_2020_2342_MOESM3_ESM.docx]

Figure S2: Scatter plot of FPKM values between replicates or genotypes. The log_10_ transformed values of replicates of WT (WTL13,WTL14, WTL15) and *PpSnRK1αoe* (OELS16, OELS17, OELS18) are plotted.
