# Supplementary material for: Overexpression of PpSnRK1α in tomato enhanced salt tolerance by regulating ABA signaling pathway and reactive oxygen metabolism
Source: BMC Plant Biol. 2020 Mar 26;20:128. doi: 10.1186/s12870-020-02342-2 (PMC7099830; doi:10.1186/s12870-020-02342-2)
Supplement: Supplementary file 6 — Additional file 6 : Table S4. GO enrichment analysis. [file 12870_2020_2342_MOESM6_ESM.docx]

Figure S2 TFs of 37 families regulated by *PpSnRK1α*
