# Supplementary material for: Overexpression of PpSnRK1α in tomato enhanced salt tolerance by regulating ABA signaling pathway and reactive oxygen metabolism
Source: BMC Plant Biol. 2020 Mar 26;20:128. doi: 10.1186/s12870-020-02342-2 (PMC7099830; doi:10.1186/s12870-020-02342-2)
Supplement: Supplementary file 9 — Additional file 9 : Table S6. primers used in this study. [file 12870_2020_2342_MOESM9_ESM.docx]

Table S2. Primers used in this study

| Gene | Accession number | Primer sequence (5’-3’) |
| --- | --- | --- |
| *SnRK1α* | ppa004347m/ Solyc03g115700.2.1 | F:GAGGTGGTTAAGATGGGAT |
|  |  | G:TCAGCTCCAAGATAGCCA |
| *PpSnRK1α* | PRUPE_3G262900 | F: GAGGTGGTTAAGATGGGAT |
|  |  | R: TCAGCTCCAAGATAGCCA |
| *SlEF1α* | Solyc06g005060.2.1 | F: TGGAAACGGATATGCCCCTG  R: TGGGCTTGGTGGGAATCATC |
| *SlPP2C37* | Solyc03g096670.3 | F: GGAGGTACACAAACTGCGGA |
|  |  | R: TTTGGAGTTAGGACGGCCAC |
| *SlPYL4* | Solyc06g050500.2 | F: GGTTCAGTCCGGTTTCCACT |
|  |  | R: ACGTGGCAGCTCTTGAGAAA |
| *SlPYL8* | Solyc01g095700.3 | F: CAGCCACCACTAGCACTGAA |
|  |  | R: TTCCCTTCAGGCACATCCAC |
| *SlNAC022* | Solyc02g061780.3 | F: GGAGTTACCTGGGATGGCAA |
|  |  | R: CCCAATCAGTTCTGGTGCCT |
| *SlNAC042* | Solyc05g021090.3 | F: AGAGGAAGTGCTGGCAAAGG |
|  |  | R: TGGAAGTTGGTGGGAGACGA |
| *SlSOD* | Solyc11g066390.1 | F: TCTTCACCACAACCAGCACT |
|  |  | R: CAGTAAGGGGTTTAGGGGTAGT |
| *SlPOD* | Solyc01g105070.2.1 | F: ACTGGCACTGAGAGAACAGC |
|  |  | R: GCGCTTGAAACTCGTCCATC |
| *SlCAT* | Solyc12g094620.1 | F: TCCTTGTCGTCCTGCTGAG |
|  |  | R: TTGATGTATCTGTCTTGCCTGTC |
